# Preliminary Exploration of the Sequence of Nerve Fiber Bundles Involvement for Idiopathic Normal Pressure Hydrocephalus: A Correlation Analysis Using Diffusion Tensor Imaging

**DOI:** 10.3389/fnins.2021.794046

**Published:** 2021-12-17

**Authors:** Wenjun Huang, Xuhao Fang, Shihong Li, Renling Mao, Chuntao Ye, Wei Liu, Guangwu Lin

**Affiliations:** ^1^Department of Radiology, Huadong Hospital Affiliated to Fudan University, Shanghai, China; ^2^Department of Neurosurgery, Huadong Hospital Affiliated to Fudan University, Shanghai, China

**Keywords:** idiopathic normal pressure hydrocephalus (INPH), diffusion tensor imaging (DTI), white matter (WM), central gray matter, dementia, gait disorder, incontinence

## Abstract

The study preliminarily explored the sequence and difference of involvement in different neuroanatomical structures in idiopathic normal pressure hydrocephalus (INPH). We retrospectively analyzed the differences in diffusion tensor imaging (DTI) parameters in 15 ROIs [including the bilateral centrum semiovale (CS), corpus callosum (CC) (body, genu, and splenium), head of the caudate nucleus (CN), internal capsule (IC) (anterior and posterior limb), thalamus (TH), and the bilateral frontal horn white matter hyperintensity (FHWMH)] between 27 INPH patients and 11 healthy controls and the correlation between DTI indices and clinical symptoms, as evaluated by the INPH grading scale (INPHGS), the Mini-Mental State Examination (MMSE), and the timed up and go test (TUG-t), before and 1 month after shunt surgery. Significant differences were observed in DTI parameters from the CS (*p*_FA1_ = 0.004, *p*_ADC1_ = 0.005) and the genu (*p*_FA2_ = 0.022; *p*_ADC2_ = 0.001) and body (*p*_FA3_ = 0.003; *p*_ADC3_ = 0.002) of the CC between the groups. The DTI parameters from the CS were strongly correlated with the MMSE score both pre-operatively and post-operatively. There was association between apparent diffusion coefficient (ADC) values of anterior and posterior limbs of the IC and MMSE. The DTI parameters of the head of the CN were correlated with motion, and the ADC value was significantly associated with the MMSE score. The FA value from TH correlated with an improvement in urination after shunt surgery. We considered that different neuroanatomical structures are affected differently by disease due to their positions in neural pathways and characteristics, which is further reflected in clinical symptoms and the prognosis of shunt surgery.

## Introduction

Idiopathic normal pressure hydrocephalus (INPH), first described by Salomón Hakim in 1965 (2016), is a chronic communicating hydrocephalus syndrome characterized by normal cerebrospinal fluid (CSF) pressure (70–200 mm H_2_O) and clinical symptoms including cognitive impairment, gait disorder, and incontinence (2016). Patients with INPH may experience different combinations and varying degrees of these typical clinical symptoms (Adams et al., 1965). It is known that the clinical symptoms of INPH can be improved by shunt surgery (Nakajima et al., 2021), but this invasive operation has uncertain efficacy and may incur some complications. Thus, understanding the neuropathological mechanisms underlying INPH might help us better select patients for surgery and predict its outcomes.

Diffusion tensor imaging (DTI), a high-resolution magnetic resonance imaging (MRI) technique combining 2D diffusion-weighted images to produce a 3D diffusion image showing the movement of water molecules in brain tissue (Siasios et al., 2016), is widely used to detect and quantify changes in white matter. White matter changes can be quantitatively measured by fractional anisotropy (FA) and the apparent diffusion coefficient (ADC), which can be used to identify small structural changes and determine the structural integrity and interstitial space of brain tissue (Le Bihan et al., 2001; Basser and Jones, 2002; Kanno et al., 2011). The ADC value is a general, quantitative measure of diffusion changes. The FA value, indicating the directivity of the ADC, reflects not only fiber microstructural damage, such as axonal degeneration or ischemic demyelination, but also the compression of white matter fibers caused by ventricular dilation, resulting in changes in fiber density and diffusion direction (Kanno et al., 2011; Kim et al., 2011). The FA value may be affected simultaneously by two different pathological processes: neural degeneration, which could decrease FA and ventricular dilation, which could increase FA (Sarica et al., 2021).

For INPH, most theories propose that ventricle dilation leads to tension and compression of the periventricular white matter, resulting in interstitial edema and progressive axonal degeneration and gliosis. On the other hand, different parts of nerve fibers are affected differently by ventricular dilation, which leads to vary changes in diffusion characteristics of different parts and different macroscopic effects (Sarica et al., 2021). Most previous studies have focused on white matter in different regions of the brain and explored differences in the influence of different white matter tracts on INPH (Kang et al., 2016; Siasios et al., 2016; Tsai et al., 2018; Younes et al., 2019). However, no definite conclusion has been drawn on the developmental sequence of related white matter injuries.

We hypothesized that in the same course of disease, the damage to different neuroanatomical structures are varied, which may be related to the location of the nerve fiber bundles and neural pathways and their own characteristics.

## Materials and Methods

### Patients

This study was approved by the Institutional Review Board of Huadong Hospital affiliated with Fudan University (Approval number: 2017K027). Informed consent was obtained from the participants before recruitment.

The study retrospectively included twenty-seven patients referred to the Department of Neurology, Huadong Hospital affiliated with Fudan University, between August 2016 and August 2020. The subjects who fulfilled the diagnostic criteria for confirmed INPH according to Experts consensus on diagnosis and treatment of normal pressure hydrocephalus in China (2016). The criteria for confirmed INPH were as follows: (1) age over 60 years; (2) at least one of the triad of symptoms (gait disturbance, dementia, and urinary incontinence) with insidious progression for more than 6 months; (3) ventricular dilatation (Evans’ index > 0.3); (4) CSF pressure <200 mm H_2_O; (5) the absence of other diseases that may account for such symptoms; and (6) a positive outcome from the CSF tap test and shunt surgery. The exclusion criteria for INPH were as follows: (1) cerebral infarction and dementia caused by recent heavy drinking, hospitalization for severe mental illness and other clear causes; and (2) secondary normal pressure hydrocephalus. The inclusion criteria for healthy elderly individuals were as follows: (1) age over 60 years; (2) no gait disorder, cognitive impairment or urination disorder, and normal MMSE score; (3) conventional cerebral MRI showing no abnormality; and (4) no active neurological, systemic or psychiatric diseases.

### Clinical Assessment

All subjects underwent detailed clinical examinations by two neurosurgeons, which involved the INPH grading scale (INPHGS), the Mini-Mental State Examination (MMSE) and, for INPH patients, and the timed up and go test (TUG-t), before shunt surgery and 1 month after shunt surgery.

The INPHGS is a clinician-rated scale used to assess the severity of INPH symptoms (cognitive impairment, gait disturbance, and urinary disturbance are each evaluated on a scale from 0 to 4) that provides a reliable and effective evaluation though interviews with patients and caregivers (Nakajima et al., 2021). The total score can be used as an index, together with the evaluation points for the MMSE and TUG-t.

A lumbar tap removing 30 ml of CSF was performed on all INPH patients. After the tap, all patients were re-evaluated using the INPHGS and the TUG test. The following criteria were used to identify positive outcome: improvement of one point or more on the INPHGS or more than 10% improvement in time on the TUG test (Kang et al., 2016). A shunt valve was inserted in the lumbar subarachnoid space in all the patients who underwent a lumboperitoneal (LP) shunt procedure.

### Magnetic Resonance Imaging Methodology and Data Analysis

All MRI examinations were performed with a 3T MRI system (MAGNETOM Prisma, Siemens AG) prior to any treatment. DTI data were obtained from all participants along 10 gradient-encoding directions with *b* values 0 and 1,000 s/mm^2^, a field-of-view (FOV) of 220 mm × 220 mm with 56 slices, a slice thickness of 2 mm with a 0.6 mm gap, an acquired resolution of 1.15 mm × 1.15 mm × 2.00 mm, a reconstructed resolution of 0.57 mm × 0.57 mm × 2.00 mm, an echo time (TE) of 78 ms, and a repetition time (TR) of 4,600 ms. The ADC and FA values were calculated using the NUMARIS/4 Siemens AG (syngo MR E11). Regions of interests (ROIs) were manually drawn on DTI images with *b*-values of 0 using a program developed in house and produced by Siemens AG.

### The Placement of Regions of Interests

The thalamic radiation, a round-trip fiber connecting the thalamus and the cortex, travels between the thalamus, caudate and lenticulate nuclei through the centum semiovale and radiates into the cerebral cortex in a fan shape. The superior and centroparietal thalamic radiations connect the frontal and parietal lobes with the ventral thalamic nuclei through the anterior and posterior limbs of the internal capsule (IC). In another way, the anatomical structure of superior thalamus radiation is close to the and diffuses around the lateral ventricle wall, so it could be affected by ventriculomegaly. The corpus callosum (CC) is adjacent to the lateral ventricle and is greatly affected by dilation of the lateral ventricle. The corticospinal tract (CST) arises from the frontal cortex and passes through the centum semiovale toward the posterior limb of the IC.

**FIGURE 1:**
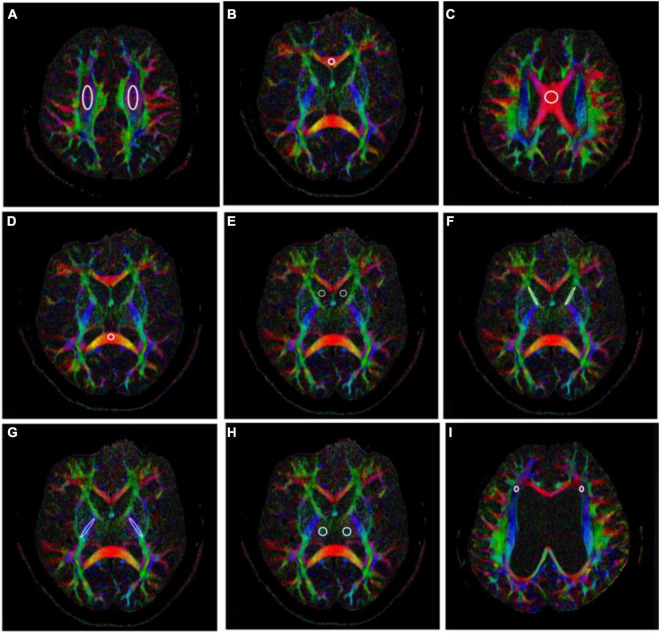
Fifteen regions of interests (ROIs) placed along the right and left centrum semiovale (CS) **(A)**, genu of the corpus callosum (CC) **(B)**, body of the CC **(C)**, splenium of the CC **(D)**, right and left head of the caudate nucleus (CN) **(E)**, right and left anterior limb of the internal capsule (IC) **(F)**, right and left posterior limb of the IC **(G)**, right and left thalamus **(H)**, and right and left frontal horn white matter hyperintensity (FHWMH) **(I)**.

According to the above, fifteen ROIs were manually placed along the right and left centrum semiovale (CS), CC (body, genu, and splenium), right and left frontal horn white matter hyperintensity (FHWMH), right and left head of the caudate nucleus (CN), right and left IC (anterior and posterior limb), and right and left thalamus (TH) (Figure 1). All ROIs were delineated by an experienced neuroradiologist.

### Statistical Analyses

Statistical analyses were conducted using the Statistical Package for Social Science, version 24.0 (SPSS, Chicago, IL, United States).

Clinical symptom statistics are expressed as the median (quartile). We compared the data between the INPH and control groups and between measurements made pre-operatively and 1 month post-operatively with the Mann-Whitney *U* test.

We used an independent-samples *t*-test to compare DTI parameters (FA and ADC) for the 15 ROIs between patients with INPH and healthy controls. An independent-samples *t*-test was also used to investigate any differences between the right and left hemispheres in these parameters. We considered the voxels to be independent of each other; therefore, no multiple comparison correction was performed. The results were deemed to be significant at a value of *p* < 0.05.

Spearman’s correlation coefficient was used to estimate the relationship between pre-operative DTI parameters and the changes between the pre-operative and post-operative clinical measures.

## Results

### Patient’s Characteristics

The 27 INPH patients [twenty-one males and six females with a median age of 74 (range 66–89) years] with symptom duration 30.48 (20.82–40.14) months and 11 healthy controls [four males and seven females with a median age of 68.36 (range 64–75) years] constituted the final sample for analysis. There were remarkable differences between the groups in their INPHGS, MMSE, and TUG-t scores. Furthermore, all INPH patients showed notable improvement in clinical measures (INPHGS, MMSE, and TUG-t) 1 month after shunting (*p* < 0.001). Evans’ index in patients with INPH was significantly higher than that in normal elderly patients before surgery (*p*1 < 0.001). and there was no significant change 1 month after shunt (*p*2 > 0.05). The evaluation results are displayed in Table 1.

**TABLE 1:** Clinical measures of the idiopathic normal pressure hydrocephalus (INPH) patients obtained pre-operatively and 1 month post-operatively and of the healthy controls.

	INPH (*n* = 27)		Healthy control, HC (*n* = 11)	*P*1-value	*P*2-value
	Pre-operative	1 month post-operative		Pre vs. HC	Pre vs. post
Age (average, range)	75.07 (66–89)		68.36 (64–75)	0.008	
Sex (male/female)	21/6		4/7	0.015*	
Symptom duration (average, range)	30.48 (20.82–40.14)				
Evans’ index	0.323 ± 0.046	0.320 ± 0.019	0.256 ± 0.033	<0.001**	0.665
**INPHGS**					
Gait	3.00 (0)	2.00 (0)	0 (0)	<0.001**	<0.001**
Cognition	3 (1)	2 (1)	0 (1)	<0.001**	0.002**
Urination	3 (1)	1 (1)	0 (0)	<0.001**	<0.001**
Total	8 (2)	5 (2)	0 (1)	<0.001**	<0.001**
Mini-Mental State Examination (MMSE)	18.15 ± 7.03	23 (5)	29 (1)	<0.001**	0.027*
Timed up and go test (TUG-t)	21.64 (8.72)	15.78 (8.13)	8.96 (1.89)	<0.001**	0.001**

*Values denote the mean ± standard deviation or median (quartile).*

Significant differences are marked with *p < 0.05 and **p < 0.01.

### Clinical Assessment Comparison

The differences between the pre-operative INPH and the healthy control clinical assessment (INPHGS, MMSE, and TUG-t) scores were significant (*p*1 < 0.001). Significant improvement was observed in the gait and urination assessments, as seen in the comparison between the pre-operative and post-operative INPH group scores (*p*2 < 0.001). The cognitive improvement was less significant than that of the improvements in gait and urination but still indicated statistical significance (*p*2′ < 0.05). There was no significant change in the Evans index after shunt surgery (*p*2″ > 0.05) (Table 1).

### Diffusion Tensor Imaging Parameter Comparison Between Pre-operative Idiopathic Normal Pressure Hydrocephalus Patients and Healthy Controls

There was no significant difference in the DTI parameters between the bilateral hemispheres in either group. Therefore, we took the mean value of the DTI parameters of the left and right hemispheres for the following intergroup and intragroup comparative analyses.

Regarding the FA value, compared with the healthy controls, the pre-operative INPH patients demonstrated a significant decrease in the CS, the genu and body of CC, and the anterior and posterior limbs of the IC (*p* = 0.004, 0.022, 0.003, 0.037, and 0.025, respectively). However, compared with the healthy controls, the pre-operative INPH patients demonstrated a significant increase in the ADC value in the same areas (*p* = 0.005, 0.001, 0.002, 0.037, and 0.010, respectively) as well as in the splenium of the CC and the head of the CN (*p* = 0.001 and 0.009, respectively) (Table 2).

**TABLE 2:** Comparison of the diffusion tensor imaging (DTI) parameters (FA and ADC) between the pre-operative idiopathic normal pressure hydrocephalus (INPH) patients and healthy controls.

DTI parameters	ROIs	Pre-operative INPH	Healthy control	*P*-value
FA	CS	0.385 ± 0.139	0.492 ± 0.043	0.004**
	Genu of CC	0.670 ± 0.120	0.861 ± 0.038	0.022*
	Body of CC	0.712 ± 0.136	0.849 ± 0.037	0.003**
	Splenium of CC	0.742 ± 0.156	0.894 ± 0.053	0.06
	FHWMH	0.200 ± 0.048	0.179 ± 0.024	0.24
	Head of CN	0.220 ± 0.033	0.198 ± 0.023	0.092
	Anterior limb of IC	0.584 ± 0.154	0.781 ± 0.052	0.037*
	Posterior limb of IC	0.746 ± 0.112	0.850 ± 0.031	0.025*
	Thalamus	0.314 ± 0.069	0.305 ± 0.041	0.162
ADC	CS	0.772 ± 0.240	0.438 ± 0.011	0.005**
	Genu of CC	0.622 ± 0.141	0.409 ± 0.016	0.001**
	Body of CC	0.626 ± 0.161	0.414 ± 0.021	0.002**
	Splenium of CC	0.571 ± 0.162	0.383 ± 0.024	0.001**
	FHWMH	0.863 ± 0.115	0.830 ± 0.071	0.069
	Head of CN	0.633 ± 0.084	0.573 ± 0.024	0.009**
	Anterior limb of IC	0.560 ± 0.177	0.373 ± 0.047	0.037*
	Posterior limb of IC	0.514. ± 0.084	0.395 ± 0.019	0.010*
	Thalamus	0.642 ± 0.092	0.541 (0.053)	0.074

*Values denote the mean ± standard deviation or median (quartile).*

Significant differences are marked with *p < 0.05 and **p < 0.01.

### Relationship Between Pre-operative Diffusion Tensor Imaging Parameters and Clinical Symptoms

#### Pre-operative Clinical Assessment

**FIGURE 2:**
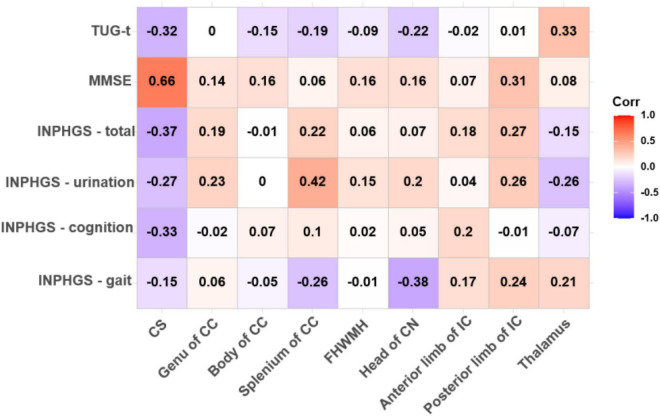
Correlations between fractional anisotropy (FA) value and pre-operative clinical evaluation score. The numbers in the cell represent *r* values.

We performed Spearman correlation analysis between the pre-operative DTI parameters and pre-operative clinical scores of all INPH patients and obtained varying degrees of correlation for the different comparisons. The mean FA of the bilateral CS demonstrated a strong and expressively significant correlation with the MMSE score (*r* = 0.659, *p* < 0.001), a moderate, negative and almost significant correlation with the INPHGS total score (*r* = −0.369, *p* = 0.058), and strong, negative and borderline significant negative correlations with the INPHGS cognitive score and the TUG-t score (*r*1 = −0.327, *p*1 = 0.096 and *r*2 = −0.323, *p*2 = 0.100, respectively) (Figure 2).

**FIGURE 3:**
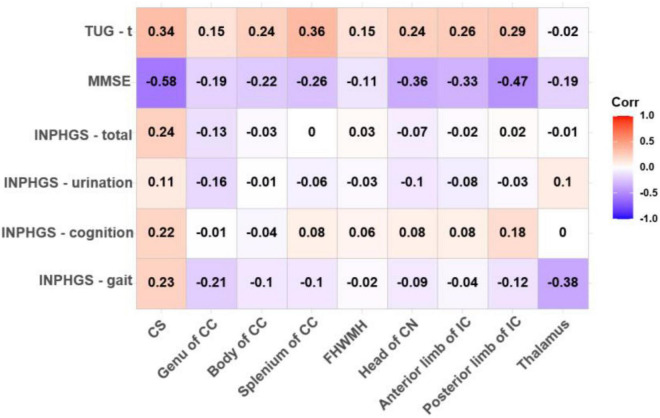
Correlations between apparent diffusion coefficient (ADC) value and pre-operative clinical evaluation score. The numbers in the cell represent *r* values.

The ADC value in the CS had a strong, negative and significant correlation with the MMSE score (*r* = −0.579, *p* = 0.002) and an almost significant correlation with the TUG-t score (*r* = 0.337, *p* = 0.085). Similarly, in the posterior limb of the IC and the thalamus, the FA values were moderately correlated with the MMSE and TUG-t scores (*r*1 = 0.314, *p*1 = 0.111; *r*2 = 0.326, *p*2 = 0.097), but these differences were not statistically significant (Figures 2, 3).

Spearman correlation analysis also revealed a moderately significant correlation between the FA value in the splenium of the CC and the INPHGS urination score (*r* = 0.424, *p* = 0.027) and an almost significant correlation between the FA value in the head of the CN and motor function (the INPHGS gait score) (*r* = −0.379, *p* = 0.051) (Figure 2).

There was a negative, nearly significant correlation between the ADC in the head of the CN (*r* = −0.357, *p* = 0.068) and in anterior limb of IC (*r* = −0.326, *p* = 0.097) and the MMSE score and a strong, negative and significant correlation between the ADC in the posterior limb of the IC and the MMSE score (*r* = −0.468, *p* = 0.014). The ADC for the splenium of the CC and the thalamus was correlated with both the TUG-t (*r* = 0.356, *p* = 0.069) and INPHGS gait scores (*r* = −0.379, *p* = 0.051), respectively. However, these correlations did not reach statistical significance (Figure 3).

There were no significant correlations between the DTI metrics in the body of the CC and FHWMH with any of the clinical evaluation scores.

### One-Month Post-operative Clinical Assessment

**FIGURE 4:**
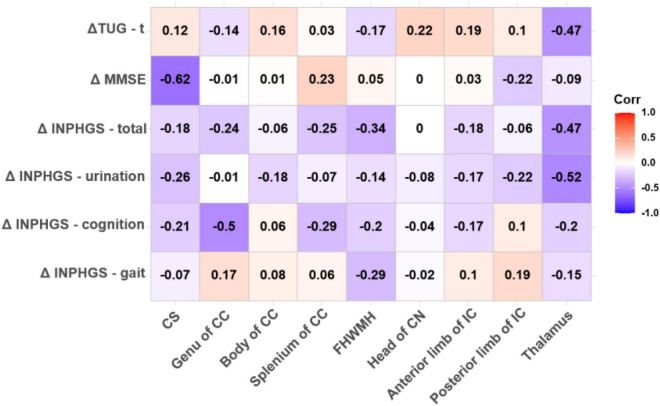
Correlation between fractional anisotropy (FA) value and changes in clinical evaluation score post-operatively. The numbers in the cell represent *r* values.

**FIGURE 5:**
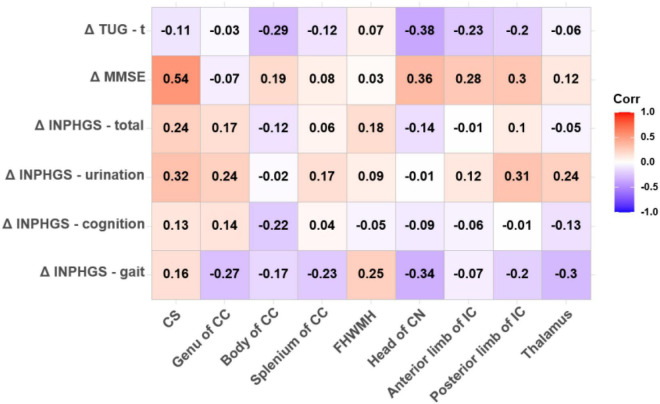
Correlation between apparent diffusion coefficient (ADC) value and changes in clinical evaluation score post-operatively. The numbers in the cell represent *r* values.

Both FA (*r* = −0.625, *p* < 0.001) and ADC (*r* = 0.530, *p* = 0.004) in the CS had strong significant correlations with the change in the MMSE score post-operatively. In the thalamus, the Spearman correlations for changes in the clinical assessment scores after shunt surgery vs. FA were also analyzed, revealing strong and significant correlations in ΔINPHGS—urination (*r* = −0.516, *p* = 0.006), ΔINPHGS—total (*r* = −0.474, *p* = 0.012), and ΔTUG-t (*r* = −0.469, *p* = 0.014) (Figures 4, 5).

A negative and significant correlation was also revealed between the ADC in the head of the CN and ΔTUG-t (*r* = −0.382, *p* = 0.050). The other correlations involving the ADC in this region were weaker, but moderate, almost significant correlations were observed with ΔINPHGS—gait (*r* = −0.338, *p* = 0.085) and ΔMMSE (*r* = 0.359, *p* = 0.066). None of the remaining correlations were significant (Figure 5).

## Discussion

This study compared the differences in DTI parameters (FA and ADC values) between INPH patients and normal elderly individuals and analyzed the correlation between pre-operative DTI parameters and pre-operative clinical scores and their post-operative changes in INPH patients. We aimed to clarify the locations where white matter or central gray matter were affected by INPH and the sequence of white matter fiber bundle involvement over the course of disease progression to explore the possible mechanism underlying the occurrence and changes in clinical symptoms in patients and establish a foundation for promoting a window for disease diagnosis and improving the evaluation of the pre-operative prognosis.

Diffusion tensor imaging can be used to evaluate the integrity of the white matter pathway by measuring the preferred direction of water diffusion in white matter fiber bundles. FA values (reflecting the degree of anisotropy) and ADC values (presenting the degree of diffusion) were defined for quantitative evaluation of nerve injury (Bouziane et al., 2019; Spotorno et al., 2019). Among them, the heterogeneity of FA value may be related to the coexistence of two different pathological processes: neural degeneration (decreasing FA), and ventricular dilation (increase FA) (Sarica et al., 2021).

In this study, it was found that the FA and ADC values of the white matter in the investigated regions of INPH patients were different from those of healthy elderly individuals except for those of the FHWMH and thalamus, with the differences in the CS being the most substantial. We also found that compared with healthy elderly participants, INPH patients had significantly lower FA values but significantly increased ADC values for the CS and the anterior and posterior limbs of the IC. Due to abnormal CSF circulation, the lateral ventricle and the Sylvian fissure dilate, and the latter resists the pressure of lateral ventricle dilation, so the ventricle dilates mainly along the *Z*-axis (which is perpendicular to the bi-commissural line). We hypothesize that the white matter in the CS is subjected to externally induced mechanical stress first (Silverberg et al., 2015; Yamada et al., 2019; Younes et al., 2019). As the disease progresses, prolonged compression of the white matter fibers leads to interstitial edema and progressive axonal loss (Younes et al., 2019), resulting in a decrease in the FA value and an increase in the ADC value. It has also been proposed that the increased ADC value of the CS is mainly due to stretching effect of enlarged ventricle along the *Z*-axis on CST, which reduces the tortuosity of nerve tract (Hattori et al., 2011; Sarica et al., 2021) and the diffusion coefficient increases with increased water diffusivity parallel to these fibers.

In addition, we found that the FA value in the CS was significantly positively correlated with the pre-operative MMSE score and negatively correlated with the improvement of the post-operative MMSE score. ADC values in the CS and anterior and posterior limbs of the IC were correlated with the pre-operative and post-operative MMSE scores. The CS and the anterior and posterior limbs of the IC are involved in the subfrontal cortical pathway. As a key node of the subfrontal cortical pathway crossed by interhemispheric, projection and association fibers, the CS is thought to be a region more responsible for chronic neuropsychiatric symptoms than other regions (Beppu et al., 2010) and may be a critical region leading to the occurrence of cognitive impairment (Skiöld et al., 2010; Zhang et al., 2011; Blasel et al., 2012). We hypothesized that a lower FA value indicateded a lower degree of white matter involvement, and it was easier to recover to the normal neuroanatomical structure after shunt surgery, thus the corresponding impaired cognitive function could be improved more easily. Regarding the correlation analysis, we showed that the ADC value of the CS was moderately correlated with the TUG score before the shunt operation. The CST starts from the precentral motor area *via* the CS and the anterior two-thirds of the posterior limb of the IC to the pedunculus cerebri and then runs down to the pons, the medulla oblongata and spinal cord, mainly controlling body movement (Park et al., 2008). Given the findings of a study on the localization of body movement in the CST (Seo et al., 2012), we considered that the white matter DTI parameters in this region are probably closely related to the motor function of the lower limbs.

Alzheimer’s disease (AD) pathology, the presence of amyloid-β (Aβ) at the brain, has been found in 25–60% of patients with INPH (Elobeid et al., 2015; Abu Hamdeh et al., 2018). It has been found that the deposition of Aβ in brain tissue affects resting state functional connectivity and thus clinical symptoms (Khoo et al., 2016; Bommarito et al., 2021), and that the CS is more susceptible to the deposition of Aβ than other regions of the white matter (Charidimou et al., 2014). We concluded that the CS may be a very important region with early involvement in the disease progression of INPH. As CSF is pathologically redistributed, the ventricular system first expands along the *Z*-axis. The resulting long-term pressure increase reduces the compliance of the upper brain tissue and limits the arterial pulse, leading to dysfunction of the brain lymphatic system (Yokota et al., 2019), which further aggravates the CSF circulation disorder. Combined with external mechanical pressure and internal metabolic abnormalities, such as decreased removal of hazardous substances (Silverberg et al., 2015), changes in the CS, which is first involved in the CST and the subfrontal cortical pathways, lead to reduced pathway connectivity and impaired integrity of the cognitive and motor networks. The most important clinical symptoms, gait and cognitive impairment, may be caused by early damage to the white matter in the CS, followed by damage involving the downstream nerve fibers along the white matter fibers (Selemon and Goldman-Rakic, 1985).

In this study, it was found that the FA and ADC values in the head of the CN in the INPH group were slightly higher than those in the control group, and they were found to be correlated with the gait and MMSE scores in both pre-operative and post-operative evaluations. Osuka et al. (2010) showed that the FA value of the CN in patients with hydrocephalus was significantly higher than that in normal elderly people and in those with obvious brain atrophy. In the brain, the FA value is affected by the diameter, density and range of nerve fiber myelin formation (Rose et al., 2012). The CN retains a relatively normal extracellular space after compression due to its stronger compression characteristics, which makes it less affected by edema (Torvik and Stenwig, 1977; Del Bigio and Bruni, 1988; DeVito et al., 2007). This may be the reason why the CN is relatively unaffected in the early stage of INPH and why the decrease in the FA value for the CN is delayed. As a part of the striatum, the head of the CN plays an important role in motor, cognitive and goal-oriented behavior (Robbins and Everitt, 1996; Middleton and Strick, 2000; Mestres-Missé et al., 2012). The anterior and middle parts of the CN connect to the prefrontal cortex, and the posterior part connects to the motor cortex, contributing to the formation of the cognitive circuit (Goldman and Nauta, 1977; Selemon and Goldman-Rakic, 1985; Lehéricy et al., 2004). Different parts of the CN are connected by longitudinal fibers, which help to integrate different cognitive functions with varying degrees of complexity (Kotz et al., 2013). Based on the above, we speculated that due to its downstream location in the nerve fiber bundle and its own characteristics, the CN sustains damage later and to a lesser degree than to other brain structures during the development of the disease. As the disease progresses, a progressive decrease in the FA value and the volume of the CN may be accompanied by a significant increase in gait and cognitive impairment.

We also found that the improvement in urination and the TUG-t score of patients after shunt surgery was significantly correlated with the thalamic FA value. Extensive white matter fiber connections between the thalamus and frontal lobes may form the neuroanatomical basis for disease progression (Black et al., 2009; Back et al., 2011; Griffanti et al., 2018). In terms of metabolism, intracranial CSF redistribution and local N-acetyl aspartate increases are associated with the decreased blood supply to the thalamus, resulting in subsequent axonal degeneration (Lundin et al., 2011). In our study, we did not find a significant correlation between thalamic DTI parameters and pre-operative clinical symptoms, which may be related to the course of disease and the degree to which the thalamus tolerates metabolic injury. The thalamus is located downstream of nerve fiber tracts and anatomically on both sides of the third ventricle. Since expansion of the third ventricle is not obvious in INPH, the mechanical pressure on the thalamus is presumed to be slight. On the other hand, due to the change in neuroplasticity and the improved efficiency of neural conductivity, the FA value of the thalamus increases in the early stage of INPH to compensate for the corresponding neural function (Mole et al., 2016; Tsai et al., 2018). Thus, we believe that damage to the thalamus mainly stems from damaged upstream white matter fibers and their intrinsic characteristics.

This study has some limitations. The first is that this was a cross-sectional study with a small sample size, so that these results should be interpreted cautiously. Further studies are needed to confirm our results. In addition, previous studies have shown that with the growth of age, FA value has a downward trend, and ADC value has a rising trend. Although ROI analysis is easy to perform, does not require third-party software and is suitable for clinical studies, there is a certain degree of subjectivity in how the ROI is outlined. Additionally, we did not subgroup the patients with diagnosed INPH according to the course of disease, so we were unable to clarify the differences in white matter integrity of different neural anatomical structures under different courses of disease. Finally, we did not conduct a review of the DTI metrics for the patients after the shunt operation, so we were unable to understand the changes in the post-operative white matter microstructure of the patients.

## Conclusion

We consider the sequence of involvement and degree of injury of different neural anatomical structures in the development of INPH disease to be caused by the coordination of three factors: external mechanical stress, the location of the nerve fiber bundle of the structure and the internal characteristics of the structure. The white matter in the CS sustains damage earliest and is most significantly affected, followed by the thalamus and the CN. When we analyzed each case in detail, we also found that the patients with the most severe pre-operative clinical symptoms and the longest course of disease had significantly lower FA values in the CS than the other patients, while there was no significant difference in the FA values from the head of the CN and the thalamus.

## Data Availability Statement

The raw data supporting the conclusions of this article will be made available by the authors, without undue reservation.

## Ethics Statement

The studies involving human participants were reviewed and approved by the Institutional Review Board of Huadong Hospital affiliated with Fudan University (Approval number: 2017K027). Written informed consent for participation was not required for this study in accordance with the national legislation and the institutional requirements.

## Author Contributions

WH, XF, SL, RM, CY, WL, and GL made a substantial contribution to the concept and design, acquisition of data or analysis, and interpretation of data. WH, XF, and SL drafted the manuscript and revised it critically for relevant intellectual content. WH and XF performed the MR examination and follow-up of patients. GL and RM were responsible for funding acquisition. All the authors approved the final version of the manuscript.

## Conflict of Interest

The authors declare that the research was conducted in the absence of any commercial or financial relationships that could be construed as a potential conflict of interest.

## Publisher’s Note

All claims expressed in this article are solely those of the authors and do not necessarily represent those of their affiliated organizations, or those of the publisher, the editors and the reviewers. Any product that may be evaluated in this article, or claim that may be made by its manufacturer, is not guaranteed or endorsed by the publisher.
